# Process Optimization for the Bioinspired Synthesis of Gold Nanoparticles Using *Cordyceps militaris*, Its Characterization, and Assessment of Enhanced Therapeutic Efficacy

**DOI:** 10.3390/ph16091311

**Published:** 2023-09-16

**Authors:** Girish Gawas, Muniappan Ayyanar, Nilambari Gurav, Dinesh Hase, Vaishali Murade, Sameer Nadaf, Mohd Shahnawaz Khan, Rupesh Chikhale, Mohan Kalaskar, Shailendra Gurav

**Affiliations:** 1 Department of Pharmacognosy, Goa College of Pharmacy, Goa University, Panaji 403001, India; girishgawas95@gmail.com; 2 Department of Botany, A.V.V.M. Sri Pushpam College (Autonomous), Affiliated to Bharathidasan University, Poondi 613503, India; asmayyanar@yahoo.com; 3 PES’s Rajaram and Tarabai Bandekar College of Pharmacy, Goa University, Ponda 403401, India; nilagurav@rediffmail.com; 4 Department of Pharmacognosy, Amrutwahini College of Pharmacy, Sangamner 422608, India; dinesh23787@gmail.com; 5 Department of Chemistry and Research Centre, Padmashri Vikhe Patil College Pravaranagar, Loni 445001, India; vaishali.hase66@gmail.com; 6 Bharati Vidyapeeth College of Pharmacy, Palus 416310, India; sam.nadaf@rediffmail.com; 7 Department of Biochemistry, College of Science, King Saud University, Riyadh 11451, Saudi Arabia; moskhan@ksu.edu.sa; 8 UCL School of Pharmacy, 29–39 Brunswick Square, London WC1N 1AX, UK; r.chikhale@ucl.ac.uk; 9 R. C. Patel Institute of Pharmaceutical Education and Research, Shirpur 425405, India; kalaskar.mohan@gmail.com

**Keywords:** green synthesis, central composite design, antioxidant activity, antidiabetic activity, process optimization

## Abstract

The promising therapeutic implications of nanoparticles have spurred their development for biomedical applications. An eco-friendly methodology synthesizes gold nanoparticles using *Cordyceps militaris*, an edible mushroom (*Cord*-Au-NPs), using a quality-by-design approach (central composite design). UV-visible spectroscopy analysis revealed an absorption peak at 540–550 nm, thus confirming the synthesis of gold nanoparticles. *Cord*-Au-NPs have a crystalline structure, as evidenced by the diffraction peaks. The zeta potential value of −19.42 mV signifies the stability of *Cord*-Au-NPs. XRD study shows gold facets and EDX analysis revealed a strong peak of spherical nanoparticles in the gold region with a mean particle size of 7.18 nm and a polydispersity index of 0.096. The obtained peaks are closely associated with phenolic groups, lipids, and proteins, as examined by FTIR, suggesting that they function as the reducing agent. *Cord*-Au-NPs exhibited dose-dependent antioxidant, antidiabetic, and antibacterial activity. The method is eco-friendly, nontoxic, less time-consuming, and does not use synthetic materials, leading to higher capabilities in biomedical applications.

## 1. Introduction

Nanotechnology is an area of research and development concerned with designing environmentally friendly synthetic methods for material production [1]. It studies nanoscale materials with 1–100 nm diameters and their applications [2]. Researchers have been intrigued by the properties of nanostructures, such as nanoparticles, nanocapsules, nanotubes, and nanoflowers, for decades because of their applications in various fields, mainly applied medical sciences [3]. Nanoparticles with improved properties have become increasingly crucial in multiple health and environmental applications. Several synthesis methods, including chemical, physical, and biological ones, have been developed to create these nanoparticles [4,5]. However, one or more potentially harmful chemical species are adsorbed on the surface of materials synthesized using chemical and physical methods. Moreover, some toxic compounds may be used as reducing and stabilizing agents in chemically mediated nanoparticle synthesis to prevent aggregation, whereas the physical process involves high temperatures and expensive materials.

Gold is mainly a valuable, inert, and nontoxic metal, and it is employed to cure various diseases [6]. Nature provides a plethora of plant resources, including a purifying technique that is low-cost, highly reproducible, environmentally benign, and exact. There has been a rise in interest in environmentally friendly methods of manufacturing gold nanoparticles, which involve using plant extract, bacteria, and mushrooms as reducing agents and stabilizers. Gold nanoparticles (Au-NPs) have been successfully developed using greener substrates such as enzymes, fungi, and algae [2]. To overcome the limitations of conventional methods, the researchers developed eco-friendly and cost-effective biological methods at room temperature that produced highly stable nanoparticles that do not flocculate even without an external chemical stabilizing agent [7].

*Cordyceps militaris* is a mushroom fungus prevalent for its beneficial effects on sick and older people and those recovering from ailments. *C. militaris* is enriched with polysaccharides, cordycepin, proteins, adenosine, ergosterol, and myriocin. It has been used to treat various diseases, including lung function, kidney function, and the immune system [8]. Polysaccharides containing α-glucose, α-mannose, α-galactose, and α-arabinose with α-type glycosidic linkage have antioxidant, immunomodulatory, hypoglycemic, and anti-inflammatory properties [9,10].

Several researchers have reported the *C.-militaris*-mediated fabrication of metallic (silver, gold, and platinum) nanoparticles and their medicinal potential [11,12,13]. Li et al. [14] proposed photolytic degradation of methylene blue using *C.-militaris*-mediated zinc oxide nanoparticles. Suksiriworapong et al. [15] reported CD44-Targeted Lipid Polymer Hybrid Nanoparticles using *C. militaris* for their enhanced anti-breast-cancer effect. Rupa et al. [16] stated the antioxidant, antimicrobial, and anti-inflammatory activity of *C.-militaris*-mediated nanoemulsion.

With their proposed mechanism, we have evidenced the *C.-militaris*-mediated eco-synthesis of zinc oxide nanoparticles and their antibacterial, antidiabetic, and antioxidant potential in our research laboratory [17]. Despite the reports on *C.-militaris*-mediated synthesis of gold nanoparticles, no one has attempted to assess the effect of process variables in their eco-synthesis. The present study demonstrated the green synthesis employing quality by design approach (central composite design), characterization, and biological assessment of gold nanoparticles mediated through an aqueous extract of *C. militaris*, i.e., *Cord*-Au-NPs.

## 2. Results and Discussion

*C. militaris* acts as a stabilizing and reducing agent, while trichlorogold, i.e., gold chloride, is a gold precursor. The aqueous solution of *C. militaris* extract (CME) was added to the trichlorogold aqueous solution. The color change indicated the reduction of gold ions into gold nanoparticles, as shown in Figure S1 (Supplementary File). The dark pink color was formed at one hour of incubation; these color changes occur due to surface plasmon vibration being excited with the gold nanoparticle synthesis.

Nanoparticles of varying sizes, shapes, and morphologies are synthesized due to variations in the composition and concentration of reducing agents in CME. Reducing metal ions into metallic nanoparticles most likely involves oxidizing the hydroxyl group of polysaccharides to the carboxyl group. Reduced metal atoms are nucleated when metal ions are first activated from their monovalent or divalent oxidation states [18].

### 2.1. UV-Visible Spectroscopy Analysis

**Figure 1:**
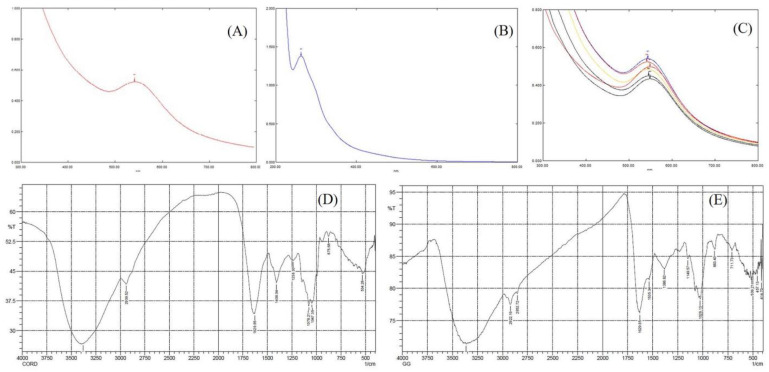
UV spectra of (**A**) Au-NPs, (**B**) CME, and (**C**) *Cord*-Au-NPs as a function of reaction time; FTIR spectra of CME (**D**) and *Cord*-Au-NPs (**E**).

The use of UV-vis spectroscopy to assess the fabrication and stability of Au-NPs in an aqueous solution is a beneficial approach. UV-vis spectrum confirms the formation of gold nanoparticles from trichlorogold exhibiting a surface plasmon resonance (SPR) absorbance band around 540–550 nm (Figure 1A–C) [2,3]. The formation of Au-NPs in the sample was evident from the change in solution color from light yellow to ruby red. As light passes through the nanoparticles in the sample, the sample absorbs specific wavelengths, and the intensity of wavelengths is reduced in the beam emitted from the sample [19]. Monitoring the reaction kinetics using UV-vis spectroscopy confirmed the completion of the reaction in the sample after 3 min, as evident from the stability, with no further significant change beyond this time (Figure 1). The concentration of generated Au-NPs with the sample was determined spectrophotometrically using the Beer–Lambert law with an extinction coefficient of 1.8 × 10^10^ M^−1^ cm^−1^ [5]. Furthermore, it gradually rises in intensity as a function of reaction time with a slight change in the peak wavelength. Therefore, it was possible to track the bioreduction of gold in an aqueous solution by diluting tiny aliquots of the sample solution with double-distilled water and subsequently measuring the solution’s UV-vis spectrum.

### 2.2. Optimization by Factorial Design Approach

#### Effect of Variables on Absorbance, Particle Size, and Zeta Potential

Absorbance, particle size, and zeta potential values for *Cord*-Au-NPs batches ranged from 0.37 to 0.554, 4.22 to 16.68 nm, and −18.96 to −8.59 mV, respectively (Table 1). The linear model was the best fit for the response Y_1_ (R^2^: 0.9561 and PRESS: 0.0024), whereas the quadratic model was the best fit for the responses Y_2_ (R^2^: 0.9959 and PRESS: 2.37) and Y_3_ (R^2^: 0.9721 and PRESS: 14.30). This confirms that the suggested model can accurately predict the 95.61%, 99.59%, and 97.21% of variances in responses Y_1_, Y_2_, and Y_3_, respectively. The models’ significance and efficacy were assessed using ANOVA (Table 2).

Small *p* (<0.05) and high F ratio values were statistically significant. The model’s F-values were determined to be 93.90, 245.39, and 34.89 for the response variables Y_1_, Y_2_, and Y_3_, respectively, highlighting the model’s significance. The model’s relevance is shown by the lack of fit values of 1.33 (Y_1_), 0.2484 (Y_2_), and 1.07 (Y_3_), which show that the models adequately fit the experimental data and various parameters significantly affect the responses. The predicted R^2^ values of 0.9158, 0.9832, and 0.8463 for Y_1_, Y_2_, and Y_3_ agreed with the adjusted R^2^ values of 0.9489, 0.9919, and 0.9443, respectively. The significance of the proposed models is illustrated by the plot of predicted against actual values (Figure S2).

The following polynomial equations illustrate the connection between independent and dependent variables:

Y_1_ (Absorbance) = +0.4689 − 0.0181 X_1_ + 0.0443 X_2_

Y_2_ (Particle size) = +12.86 − 0.9333 X_1_ + 0.5733 X_2_ − 2.24 X_1_X_2_ + 0.4756 X_1_^2^ − 1.92 X_2_^2^

Y_3_ (Zeta potential) = −13.69 − 1.36 X_1_ + 0.8650 X_2_ − 2.61 X_1_X_2_ + 0.5810 X_1_^2^ − 0.8252 X_2_^2^

**Figure 2:**
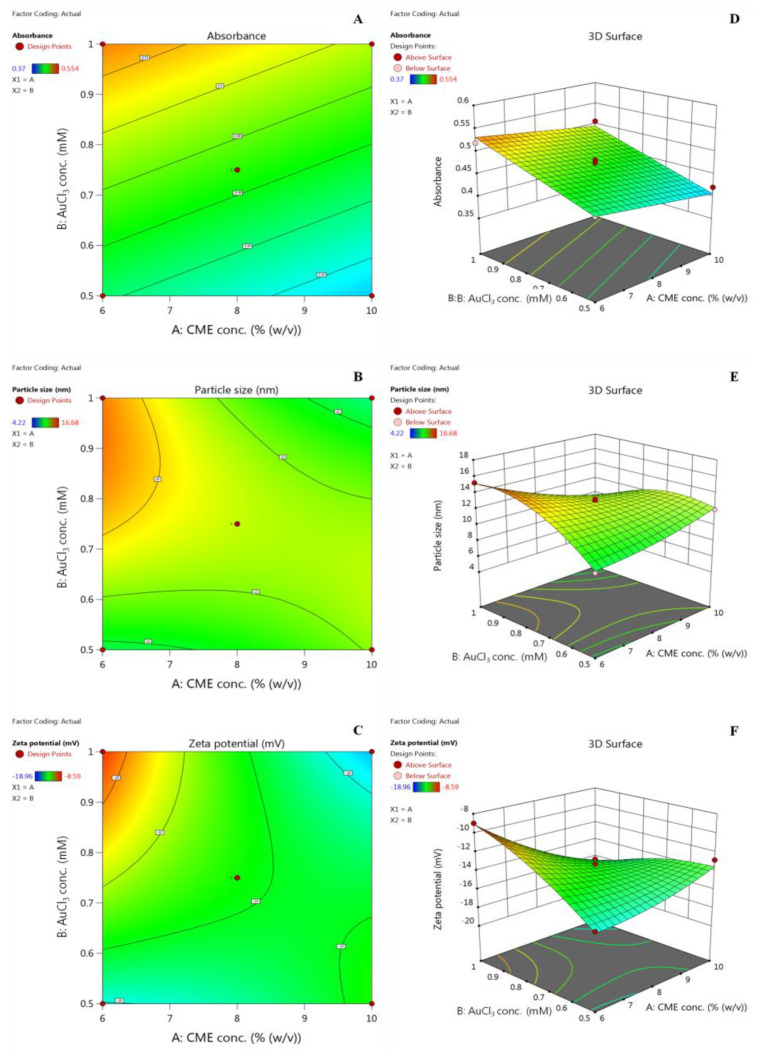
Two-dimensional contour plots (**A**–**C**) and three-dimensional response surface plots (**D**–**F**) show independent variables' effect on response variables.

Main terms X_1_ and X_2_ significantly affected response Y_1_, whereas X_1_, X_2_, X_1_X_2_, X_1_^2^, and X_2_^2^ showed significant effects on responses Y_2_ and Y_3_. Factors X_1_ and X_2_ negatively and positively affected all the studied responses. These results were further validated by 2D contour and 3D response surface plots (Figure 2). Each variable’s qualitative impact on each response parameter can be seen by carefully examining these charts. Interaction term X_1_X_2_ showed antagonistic effects on Y_2_ and Y_3_. The examination of interaction plots further supported this. Crossed lines in the plots (Figure S3) confirm interactions, while parallel lines indicate no interactions. Figure S4 shows model diagnosis plots, including a normal and residual versus run plot. A close look at the graphs reveals that the residuals typically follow a straight line, indicating that the errors are evenly distributed. The residuals vs. run plot also revealed a random dispersion.

### 2.3. Model Validation

The optimal range for the formulation parameters was determined using a graphical optimization strategy based on the desirability function approach and an overlaid contour plot. The optimal concentrations of CME and AuCl_3_ for obtaining Au-NPs are displayed in the region marked in Figure S5. Optimal values of the synthesis variables under investigation were also determined using various optimization techniques. The results demonstrated that 10% (*w*/*v*) CME concentration and AuCl_3_ at 1 mM concentration would offer Au-NPs with desired characteristics. Au-NPs were prepared using optimal values of independent variables and then examined concerning the investigated response variables. Predicted values for the absorbance, particle size, and zeta potential of the synthesized Au-NPs were 0.495, 8.82 nm, and −17.04 mV, respectively. The measured experimental values for the absorbance, particle size, and zeta potential were 0.512, 7.18 nm, and −19.42 mV, respectively. Predicted and actual experimental findings did not differ much, indicating the model’s validity.

### 2.4. Fourier Transform Infrared (FTIR) Spectroscopy

FTIR analyzed the functional groups of CME and *Cord*-Au-NPs bioactives attached to the surface of the nanoparticles, where 4000–400 cm^−1^ was the scanning range. The biomolecules potentially accountable for stabilizing the gold nanoparticles were determined using FTIR analysis. The FTIR spectra of CME (Figure 1D) show a strong absorption band at 3381.21 cm^−1^ associated with the stretching vibration of -OH and -NH, related to the H_2_O molecules and amino groups. Absorption bands at 2939.52 cm^−1^ and 1629.85 cm^−1^ correspond to -CH_2_ groups in lipids and protein C=O stretching vibrations, respectively. Phenolic group vibrations were allocated to the 1408.04 cm^−1^ absorption band. The C-O-C and C-OH stretching vibrations of aromatic acid ester and phenolics are shown in the absorption band at 1238.30 cm^−1^. At 1078, 1047, and 875 cm^−1^, β-glucans were found to have β-glycosidic linkages that were characterized by absorption peaks [17].

The FTIR spectra of *Cord*-Au-NPs (Figure 1E) show absorption bands at 3361.88 cm^−1^, 2922.16 cm^−1^, 1619.85 cm^−1^, 1149.57 cm^−1^, and 1026.13 cm^−1^, which can be allocated to -OH and N-H groups of water molecules and amino groups, -CH_2_ groups in lipids, C=O stretching of proteins, C-O-C and C-OH groups of aromatic acid ester and phenolics, respectively, and β-glycosidic bonds of β-glucans. The decrease in intensity at the above absorption peaks in *Cord*-Au-NPs signifies the involvement of the phenolic group, lipids, and proteins in the reduction process. Furthermore, they essentially act as capping agents due to the presence of different groups of compounds, providing stability and preventing aggregation of Au-NPs. The *Cord*-Au-NPs exhibit a strong binding affinity to various functional groups, as reported in the CME, a layer covering Au-NPs.

According to the FTIR spectral analysis, the stabilization and reduction of the Au-NPs occur with the involvement of different functional groups of biomolecules reported in the extract used in the study. It is well known that phytochemicals act as stabilizing agents in the fabrication of metal nanoparticles [20]; further validation is essential to identify the active molecules that are responsible for Au-NP synthesis.

### 2.5. Morphological Analysis

**Figure 3:**
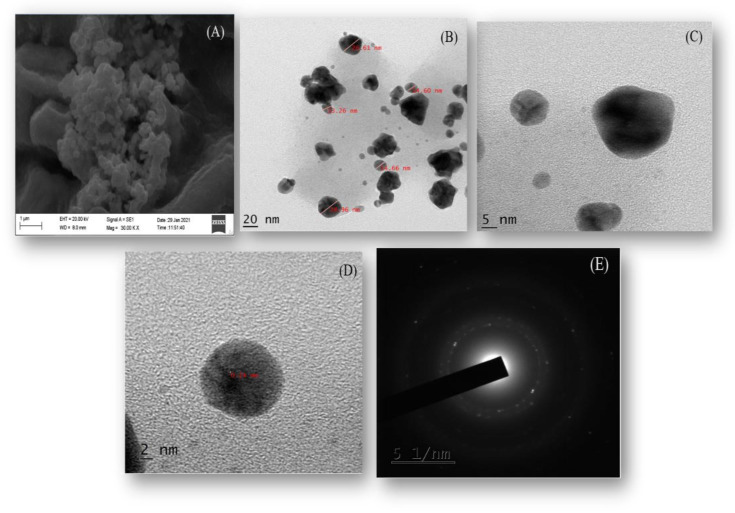
(**A**) SEM, (**B**–**D**) HRTEM, and (**E**) SAED pattern of *Cord*-Au-NPs.

The scanning electron microscope (SEM) images of the nano-sized *Cord*-Au-NPs describe the larger surface area. The SEM (Figure 3A) and high-resolution transmission electron microscope (HRTEM) (Figure 3B–D) images at different magnification levels were used to analyze the morphology of the *Cord*-Au-NPs. They revealed a cluster of predominantly spherical, irregular-shaped particles.

In addition, the selected area electron diffraction (SAED) (Figure 3E) pattern specified the polycrystalline nature of the *Cord*-Au-NPs [17]. Figure 3A illustrates the *Cord*-Au-NPs synthesized as standard protocol and shows spherical morphology with low distribution.

### 2.6. Particle Size and Zeta Potential Analysis

**Figure 4:**
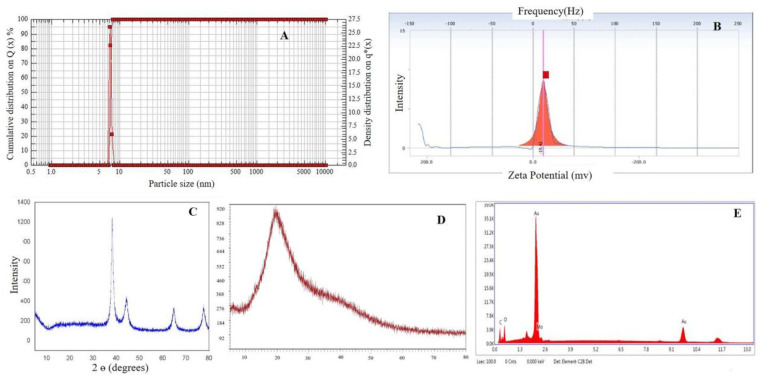
Particle size (**A**), zeta potential (**B**), XRD (**C**,**D**), and EDX (**E**) analysis of *Cord*-Au-NPs.

The size of *Cord*-Au-NPs varies from 1 to 100 nm, with an average particle size of 7.18 nm and polydispersity index (PDI) of 0.096 (Figure 4A). Nanoparticles’ net surface charge determines their zeta potential. Nanoparticles resist each other in solution, causing a Coulomb explosion; thus, they do not agglomerate. Nanoparticles with zeta potential scaling from +30 mV to −30 mV are considered stable [18,21]. *Cord*-Au-NPs had a zeta potential of −19.42 mV (Figure 4B). Zeta potential values are negative, indicating that the capping agents effectively stabilize nanoparticles by preventing agglomeration through intense negative charges. Zeta potential values are used as a hallmark indication of the stability of colloidal particles, and absolute values replicate the net electrical charge on the particles’ external surface that arises from the surface functional groups [5].

### 2.7. X-ray Diffraction (XRD) Analysis

XRD test is used to determine the size and evaluate the crystalline structure of the synthesized samples and provides information about the crystallinity of the NPs [19]. It is clear from the XRD pattern (Figure 4C,D) that the gold facets (111), (200), (220), and (311) are all represented by peaks with 2θ values between 38.26, 44.23, 64.75, and 77.67. Au-NPs synthesized using *Turbinaria conoides* reported similar observations [7]. As can be seen from the XRD pattern, the gold nanoparticles synthesized are crystalline. Debye–Scherrer equation, i.e., d = kλ/(β Cosθ), was used and revealed an average crystalline size of 6.72 nm. The reported peak values of Cord-Au-NPs also matched the planes and face-centered cubic structures of Au-NPs prepared by other green synthesis methods by various researchers [22,23,24].

### 2.8. Energy-Dispersive X-ray Analysis

Due to SPR, metallic NPs have a typical optical absorption peak of around 2.15 keV [17,25,26]. The energy-dispersive X-ray (EDX) analysis (Figure 4E) of *Cord*-Au-NPs illustrated a strong peak in the gold region (around 2.15 keV) that validated the synthesis of Au-NPs. All additional signals may have been caused by elements present in CME, as shown by the EDX data’s carbon grid signal.

### 2.9. Brine Shrimp Lethality Assay

The brine shrimp lethality experiment was used to test the cytotoxicity of biogenetically synthesized *Cord*-Au-NPs at varying doses for 24 h. Brine shrimps were highly vulnerable to *Cord*-Au-NPs, even at relatively low concentrations (Table S2) (Supplementary File). *Cord*-Au-NPs exhibited an LC_50_ value of 192.23 ± 57.34 µg/mL; given that the LC_50_ is between 100 and 1000 ppm, they may have some biological activity. After 24 h of exposure to *Cord*-Au-NPs, concentration-dependent death in brine shrimp was observed. Hitherto, the green synthesized *Cord*-ZnO-NPs using *C. militaris* and rutin-conjugated titanium dioxide nanoparticles (Rut-TiO_2_NPs) have evidenced cytotoxic effects on brine shrimps [25].

### 2.10. In Vitro Antidiabetic Activity

**Figure 5:**
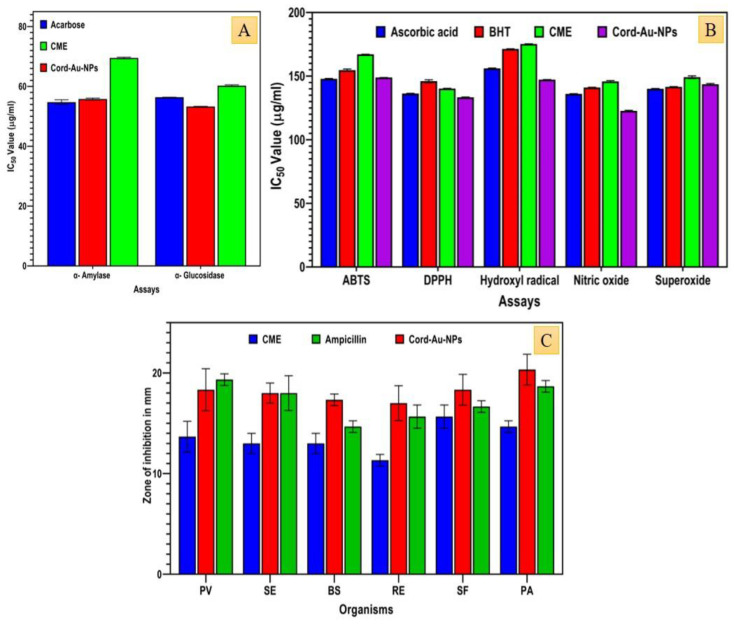
Antidiabetic ((**A**) α-amylase and α-glucosidase enzyme inhibition assays), antioxidant ((**B**) DPPH, ABTS, superoxide, nitric oxide, and hydroxyl radical scavenging assays), and antibacterial activity of CME and *Cord*-Au-NPs (PV—*Proteus vulgaris*, SE—*Staphylococcus epidermidis*, BS—*Bacillus subtilis*, RE—*Rhodococcus equi*, SF—*Shigella flexneri*, PA—*Pseudomonas aeruginosa*).

The α-amylase and α-glucosidase inhibitory activity test results showed that *Cord*-Au-NPs exhibited a higher inhibitory effect than CME (Figure 5A) and gave equivalent results to the standard acarbose used in the study.

Significant α-glucosidase enzyme inhibition (53.27 ± 0.07 µg/mL) was observed in *Cord*-Au-NPs compared to the standard acarbose (56.40 ± 0.09 µg/mL). Likewise, *Cord*-Au-NPs showed a notable α-amylase inhibitory effect (55.78 ± 0.33 µg/mL), which is comparable to the results of standard (54.74 ± 0.80). Previously, Nigella-sativa-mediated Au-NPs showed effective antidiabetic activity by inhibiting α-amylase (81% at 500 µg/mL) and α-glucosidase (82.3% at 500 µg/mL) enzymes [27]. Furthermore, oral administration of Fritillaria-cirrhosis-mediated Au-NPs possessed good antidiabetic potential in STZ-induced diabetic rats with appreciable restoration of serum, hepatic, and renal markers to normal by Au-NPs treatment [28]. *C. militaris*-mediated ZnO nanoparticles also exhibited a remarkable inhibitory effect against the enzymes α-amylase (IC_50_ of 46.29 ± 0.49 µg/mL) and α-glucosidase (47.21 ± 0.19 µg/mL), with *C. militaris* having a moderate enzyme inhibitory effect [17,25].

### 2.11. In Vitro Antioxidant Activity

The antioxidant potential of CME and Cord-Au-NPs was determined by DPPH, ABTS, superoxide, NO, and hydroxyl radical assays. All the tested samples revealed dose-dependent radical scavenging activity (Figure 5B). *Cord*-Au-NPs exhibited the highest ABTS radical scavenging activity (148.90 ± 0.05 µg/mL). A maximum hydroxyl radical scavenging effect was observed in *Cord*-Au-NPs with an IC_50_ of 147.27 ± 0.08 µg/mL. The maximum DPPH radical scavenging activity was observed in *Cord*-Au-NPs (133.29 ± 0.31 µg/mL), lower than the standard ascorbic acid and higher than the BHT. At the same time, moderate DPPH scavenging activity was recorded in CME (140.15 ± 0.29 µg/mL). According to Milanezi et al. [29], quercetin-capped Au-NPs showed a higher antioxidant potential on DPPH, ABTS, and NO radical scavenging assays. Similar findings were also reported by Zayadi et al. [30]. They observed significant antioxidant (DPPH free radical scavenging potential of 55.1% and hydroxyl radical scavenging of 50.0%) activity in *Zingiber officinale*-mediated synthesis of colloidal biogenic Au-NPs.

For the superoxide free radical scavenging assay, CME (149.19 ± 1.03 µg/mL) exhibited moderate effect and *Cord*-Au-NPs (143.56 ± 0.65 µg/mL) showed excellent free radical scavenging activity when compared to the standard, ascorbic acid (140.01 ± 0.29 µg/mL), and BHT (141.56 ± 0.22 µg/mL) (Figure 5B). Nitric oxide scavenging effect of the CME was found to be 145.85 ± 0.81 µg/mL. In contrast, the scavenging value was determined to be superior in *Cord*-Au-NPs, with an IC_50_ of 122.94 ± 0.63 µg/mL, which was recorded as a better activity than the ascorbic acid (136.02 ± 0.19 µg/mL) and BHT (141.06 ± 0.23 µg/mL). Similarly, Sathishkumar et al. [31] have demonstrated that the biosynthesis of Au-NPs with *Couroupita guianensis* fruits showed potential free radical scavenging activity on DPPH, reducing power assay, hydroxyl radical scavenging, and superoxide radical scavenging assays. Lydia et al. (2020) [32] reported that Au-NPs synthesized from *Punica granatum* revealed significant DPPH (23.6 ± 1.5 to 62.5 ± 1.8%) and hydrogen peroxide (21.6 ± 1.3 to 62.8 ± 1.8%) radical scavenging activity. Similarly, the calcinated form of the titanium oxide nanoparticles exhibited considerable free radical scavenging abilities using assays such as DPPH (IC_50_ of 95.32 ± 0.43), NO (IC_50_ of 110.03 ± 0.99 μg/mL), superoxide (IC_50_ of 136.27 ± 0.63 μg/mL), and ABTS method (IC_50_ of 119.11 ± 0.94 μg/mL) [17,25].

### 2.12. In Vitro Antibacterial Activity

**Figure 6:**
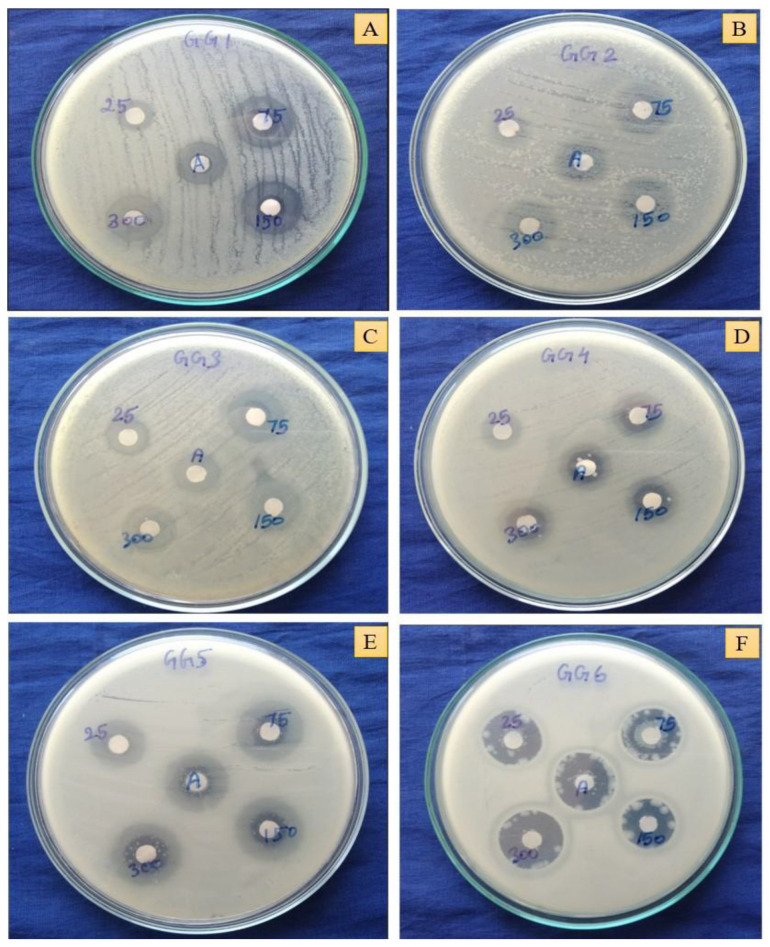
Zone of inhibition results for the *Cord*-Au-NPs. (**A**)—*Proteus vulgaris*, (**B**)—*Staphylococcus epidermidis*, (**C**)—*Bacillus subtilis*, (**D**)—*Rhodococcus equi*, (**E**)—*Shigella flexneri*, (**F**)—*Pseudomonas aeruginosa*.

*Cord*-Au-NPs showed a strong antibacterial effect by inhibiting the growth of studied bacteria at 300 µg/mL concentration (Figure 5C). *Cord*-Au-NPs exhibited a higher inhibitory effect against the growth of *Pseudomonas aeruginosa* MTCC 1748 (20.33 ± 1.52 mm), *Proteus vulgaris* MTCC 426 (18.33 ± 2.08 mm), *Staphylococcus epidermis* MTCC 435 (17.33 ± 2.08 mm), and *Shigella flexneri* MTCC 1457 (16.66 ± 0.57 mm) (Figure 6), whereas *Bacillus subtilis* MTCC 441 and *Rhodococcus equi* MTCC 2558 were most sensitive for CME. *Cord*-Au-NPs were observed to have the potential to pierce bacterial cell walls and substantially destroy cell membranes.

In agreement with our results, *Alternanthera bettzickiana* leaf-extract-mediated biocompatible Au-NPs exhibited significant antimicrobial activity against *B. subtilis* (14 ± 0.15 mm), *S. aureus* (16 ± 0.88 mm), *S. typhi* (16 ± 0.44 mm), *P. aeruginosa* (14 ± 0.58 mm), *Micrococcus luteus* (22 ± 0.44 mm), and *Enterobacter aerogenes* (06 ± 0.15 mm) [33]. Likewise, *Halymenia dilatata*-mediated biogenic synthesized Au-NPs exhibited significant antibacterial activity, with the highest zone of inhibition (21 mm) at 100 μg/mL against the selected pathogenic bacteria [34]. Bacterial drug susceptibility was assessed using the MIC value, which was also vital for testing the efficacy of antimicrobial compounds [35]. In the present investigation, *Cord*-Au-NPs showed a good inhibitory effect against the growth of *B. subtilis* and *P. vulgaris*, with an MIC of 23.44 µg/mL, followed by *P. aeruginosa* and *S. flexneri* (46.88 µg/mL) (Table S1). A moderate inhibitory effect was observed against *S. epidermis* and *S. flexneri* growth, with an MIC of 93.74 µg/mL. Likewise, CME showed potential inhibition against *P. vulgaris*, with an MIC of 46.80 µg/mL, followed by *R. equi* and *P. aeruginosa* (93.75 µg/mL). Similar results were reported by Nagalingam et al. [33] in the pathogenic bacterial strains *B. subtilis*, *S. aureus*, *M. luteus*, *E. aerogenes*, *S. typhi*, and *P. aeroginosa*, with the MIC of 10–40 µg/mL with Au-NPs synthesized from *A. bettzickiana* leaf extracts.

### 2.13. Cytotoxicity of Cord-Au-NPs

Cytotoxicity assay of *Cord*-Au-NPs inhibited 81.1% and 76.4% of the growth of Human Breast Cancer (MDA-MB-31) and Human Colon Adenocarcinoma (HT-29) cell lines at 90 μg/mL.

**Figure 7:**
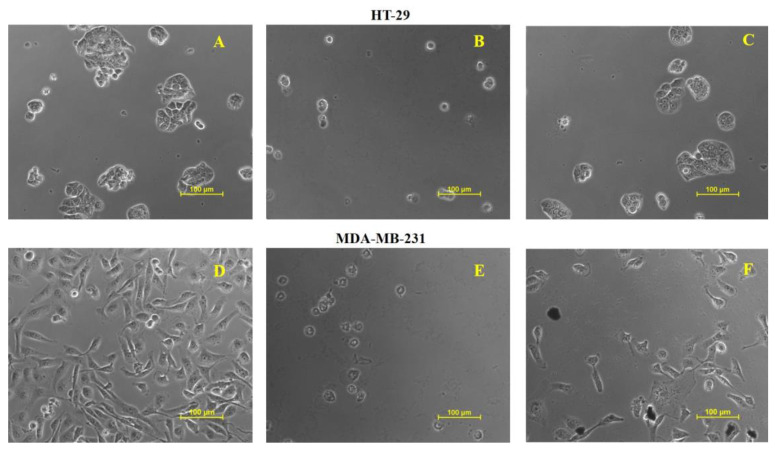
Cytotoxicity studies of test drugs ((**A**)—HT-29-Control, (**B**)—ADR, (**C**)—*Cord*-Au-NPs, (**D**)—MDA-MB-231 Control, (**E**)—ADR and (**F**)—*Cord*-Au-NPs).

Figure 7 depicts the significant growth inhibition of *Cord*-Au-NPs (Figure 7C,F) against selected cell lines compared to control (Figure 7A,D) and Adriamycin (ADR) (Figure 7B,E). Moreover, Hosny et al. [36] revealed the cytotoxicity effect of Au-NPs using MTT assay with higher efficiency in inhibiting the growth and proliferation of human breast cancer cells.

## 3. Materials and Methods

### 3.1. Chemicals

Analytical-grade Trichlorogold (AuCl_3_, 99%) was purchased from Sigma-Aldrich, St. Louis, MO, USA. All other chemicals used were of analytical grade.

### 3.2. Preparation of Test Material

As per our earlier reports, the fruiting bodies of *C. militaris* were collected from the Department of Biotechnology—Technology Incubation Centre, Bodoland University, Assam, India, followed by their microscopic and macromorphological examinations to aid their identification and confirmation [17]. A total of 10 g of freeze-dried *C. militaris* powder was added to 50 mL of double distilled water and boiled at 60 °C for 15 min. The obtained *C. militaris* extract was filtered through Whatman No.1 filter paper and stored at 4 °C until further use.

### 3.3. Cord-Au-NPs Synthesis

*Cord*-Au-NPs were synthesized following a reported method [2]. Briefly, 10 mL of CME was added to 100 mL of 1 mM gold chloride solution in a conical flask and kept in the dark at room temperature. The color change was visually observed, and the time taken for the color to change was noted. Samples were drawn routinely and analyzed spectrophotometrically using a UV-visible Spectrophotometer (Shimadzu, UV-2700, Kyoto, Japan) to verify the synthesis of *Cord*-Au-NPs.

#### 3.3.1. Optimization by Factorial Design Approach

The synthesis technique for *Cord*-Au-NPs was optimized utilizing a central composite design (CCD) (Design-Expert software V13.0; Stat-Ease Inc., Minneapolis, MN, USA). A total of 11 runs (R1–R11) were produced using software and the effects of independent variables were evaluated, namely, X_1_: CME concentration (% (*w*/*v*)) and X_2_: AuCl_3_ concentration (mM) on Y_1_: absorbance, Y_2_: particle size (nm), and Y_3_: zeta potential of prepared Au-NPs. Table 2 shows all independent and response variables and their coded and actual levels. As shown in Table 2, 11 experimental runs were generated with five distinct levels for both independent variables.

The experimental runs’ center point (X_1_ = 8% *w*/*v* and X_2_ = 0.75 mM) was analyzed in triplicate to reduce pure error. Model suitability was decided based on the coefficient of determination (R^2^), predicted error sum of squares (PRESS), and lack of fit analysis. The significance of the model was also tested with analysis of variance (ANOVA). Effects of independent variables on responses were visualized using two contour plots and 3D surface plots.

#### 3.3.2. Model Validation

Graphical optimization was utilized to achieve the optimized values for the CME concentration and X_2_: AuCl_3_ concentration that can offer Au-NPs of desired attributes, i.e., minimum particle size, zeta potential, and maximum absorbance. Finally, checkpoint analysis was performed at ideal synthesis conditions to validate the model and obtain an optimized experimental procedure.

### 3.4. Physicochemical Characterization

The synthesized *Cord*-Au-NPs were subjected to physicochemical characterization [2,17,20] using Fourier transform infrared (FT-IR) spectrophotometer (Make: Shimadzu, IR Affinity-1), an X-ray diffractometer (XRD) (Rigaku, Ultima IV, Tokyo, Japan), a scanning electron microscope with energy-dispersive X-ray (EDX) (Carl ZEISS EVO-18) analyzer, high-resolution transmission electron microscope (HRTEM) (Make: Joel, Model: JEM 2100), and particle size and zeta potential analyzer (Nanophox, NX0088—Sympatec, Clausthal-Zellerfeld, Germany). The detailed procedures for the physicochemical evaluation of green synthesized *Cord*-Au-NPs are given in the Supplementary File.

### 3.5. Therapeutic Investigation

#### 3.5.1. Brine Shrimp Lethality Bioassay

*Cord*-Au-NPs, at various concentrations, were tested for cytotoxicity using the Brine Shrimp Lethality Bioassay with *Artemia salina* eggs, as described in the literature [17,20].

#### 3.5.2. Antidiabetic Activity

The antidiabetic potential of *Cord*-Au-NPs was ascertained using in vitro enzyme (α-amylase and α-glucosidase) inhibitory assays [17,20]. Starch solution (1%) was used as a substrate for α-amylase and p-nitrophenyl-α-D-glucopyranoside (PNPG) for α-glucosidase inhibitory assays, with acarbose as an antidiabetic reference.

For the α-amylase inhibitory assay, 100 µL of the different concentrations of CME and *Cord*-Au-NPs (3.125, 6.25, 12.5, 25, 50, 100, and 200 µg/mL) were added with 1% starch solution in 20 mM phosphate buffer as the substrate solution. The mixture was incubated at 25 °C for 10 min, and then 100 µL of porcine pancreatic α-amylase enzyme (0.5 mg/mL) was added, followed by incubating the mixture for 10 min at 25 °C. To this, 200 µL of dinitro salicylic acid reagent was added to terminate the reaction. Then, the mixture was again incubated at 100 °C for 5 min. After cooling, the absorbance of samples was measured at 540 nm [10].

For the α-glucosidase inhibitory assay, 50 μL of CME and *Cord*-Au-NPs in different concentrations (3.125, 6.25, 12.5, 25, 50, 100, and 200 µg/mL) was added with 100 mM sodium phosphate buffer and 50 μL of 5 mM p-nitrophenyl-α-d-glucopyranoside solution and the mixture was incubated at 37 °C for 5 min. To this mixture, 100 μL of phosphate buffer with 0.1 U/mL α-glucosidase enzyme was added, and after 30 min of incubation, absorbance was measured at 405 nm.

The α-amylase and α-glucosidase enzyme inhibitory effects of CME and *Cord*-Au-NPs were calculated using the following formula:

% inhibition = [(Control Abs − Sample Abs)/Control Abs] × 100

#### 3.5.3. Antioxidant Activity

Antioxidants play a vital role in the treatment and management of several diseases. Hence, the antioxidant activity of *Cord*-Au-NPs was evaluated using different in vitro assays (2, 2-diphenyl-1-picrylhydrazyl (DPPH) radical, nitric oxide (NO) radical, superoxide radical, hydroxyl radical, and 2, 2′-azino-bis-(3-ethylbenzo-thiozoline-6-sulfonic acid) diammonium salt (ABTS) scavenging assay). These methods assess free radical inhibition based on the endpoints, radical generation, and reproducibility. In all these assays, CME and *Cord*-Au-NPs were prepared using 31.25, 62.5, 125, 250, and 500 µg/mL concentrations by adding respective reagents followed by the standard methodologies [17,20].

#### 3.5.4. Antibacterial Activity

Antibacterial activity of CME and *Cord*-Au-NPs were studied against the Gram-positive and Gram-negative bacterial strains *Proteus vulgaris* MTCC 426, *Staphylococcus epidermis* MTCC 435, *Bacillus subtilis* MTCC 441, *Rhodococcus equi* MTCC 2558, *Shigella flexneri* MTCC 1457, and *Pseudomonas aeruginosa* MTCC 1748 by agar disc diffusion and microdilution methods [17,20]. Different concentrations (25, 75, 150, and 300 µg/mL) were used in the agar disc diffusion assay with amoxicillin as standard (1 mg/mL). The Mueller–Hinton (MH) agar medium inoculated with a loop of each tested bacterium was used as a working culture. The prepared discs were soaked in test solutions and placed on the plates with inoculated agar and bacterial strains before incubating at 37 °C for 24 h. Then, the diameter (mm) of the inhibition zone (growth zone of bacterial strains around the discs) was measured to analyze the antibacterial effect of CME and *Cord*-Au-NPs.

In the microdilution assay, the minimal concentration at which the growth of bacteria was inhibited is considered as minimum inhibitory concentration (MIC). The bacterial strains in the above assay were used to determine the MIC in 96-well microtiter plates. Briefly, 50 µL of CME and *Cord*-Au-NPs were poured into each well at different concentrations (500–0.488 mg/mL in two-fold serial dilution) with 50 µL of MH broth. A total of 50 µL of bacterial inoculum at 106 CFU/mL was added to each well and then incubated at 37 °C for 24 h. Then, 20 µL of *p*-iodonitro tetrazolium dye (INT) at a concentration of 0.5 mg/mL was added to each well and again incubated at 37 °C for 30 min with amoxicillin (30–0.029 mg/mL) as standard. The change in color after adding the INT dye embodies the MIC of CME and *Cord*-Au-NPs.

#### 3.5.5. Anticancer Activity

Cell lines, i.e., Human Breast Cancer (MDA-MB-31) and Human Colon Adenocarcinoma (HT-29), were procured from the National Centre for Cell Science (NCCS), Pune, India. Anticancer activity of *Cord*-Au-NPs was appraised against selected cell lines using Sulforhodamine B (SRB) assay at various concentrations, i.e., 10 μg/mL, 20 μg/mL, 40 μg/mL, and 80 μg/mL [24]. The percent growth inhibition was calculated using the following formula:

[Ti/C] × 100%

where Ti—test drug concentration; C—control growth.

## 4. Conclusions

The biosynthesis of gold nanoparticles was carried out using an aqueous extract of *C. militaris*. UV-vis spectral analysis was used to validate the formation and stability of nanogold. The synthesized nanoparticles were used to characterize TEM, SEM, PSA, zeta potential analysis, ED-X-ray studies, XRD, and FTIR analysis. The gold nanoparticles possess significant antidiabetic, antioxidant, and antibacterial activities. Cytotoxicity study also confirmed the nontoxicity of *Cord*-Au-NPs. The current approach of edible-mushroom-mediated synthesis of nanoparticles deliberates the improved therapeutic potential and *Cord*-Au-NPs as a potential source of biomedical applications. This adapted synthesis method is simple, effective, environmentally friendly, nontoxic, and labor-saving.

## Figures and Tables

**Table 1:** ANOVA results for responses Y_1_ and Y_2_.

Source	Response Y_1_				Response Y_2_				Response Y_3_			
	Coeff.	Sum of Squares	F-Value	*p*-Value	Coeff.	Sum of Squares	F-Value	*p*-Value	Coeff.	Sum of Squares	F-Value	*p*-Value
**Model**	0.4689 ^a^	0.0274	93.9	<0.0001 ^b^	12.86 ^a^	140.32	245.39	<0.0001 ^b^	−13.69 ^a^	90.47	34.89	0.0007 ^b^
X_1_ -	−0.0181	0.0039	26.88	0.0008	−0.9333	10.45	91.41	0.0002	−1.36	22.09	42.59	0.0013
X_2_ -	0.0443	0.0235	160.93	<0.0001	0.5733	3.94	34.49	0.002	0.865	8.98	17.31	0.0088
X_1_X_2_					−2.24	19.98	174.72	<0.0001	−2.61	27.35	52.75	0.0008
X_1_^2^					0.4756	4.37	38.17	0.0016	0.581	6.52	12.57	0.0165
X_2_^2^					−1.92	70.92	620.17	<0.0001	−0.8252	13.14	25.35	0.004
**Residual**		0.0012				0.5718				2.59		
Lack of Fit		0.0009	1.33	0.4886 ^c^		0.1552	0.2484	0.8586 ^c^		1.6	1.07	0.5175 ^c^
Pure Error		0.0002				0.4166				0.9978		
**Cor Total**		0.0286				140.89				93.06		

^a^ Intercept; ^b^ significant; ^c^ non-significant.

**Table 2:** CCD matrix with coded and actual levels of independent and dependent variables.

Independent Variables				Levels				
			−α	Low (1)	Medium (0)		High (+1)	+α
X_1_: CME conc. (% *w*/*v*)			4	6	8		10	12
X_2_: AuCl_3_ conc. (mM)			0.25	0.5	0.75		1	1.25
**Dependent variables**				**Goal**				
Y_1_: Absorbance				Maximize				
Y_2_: Particle size (nm)				Minimize				
Y_3_: Zeta potential (mV)				Minimize				
**Std**	**Run**	**X_1_: CME Conc. (% *w*/*v*)**	**X_2_: AuCl_3_ Conc. (mM)**	**Y_1_:** **Absorbance**		**Y_2_: Particle size** **(nm)**		**Y_3_: Zeta potential (mV)**
11	1	8	0.75	0.461		13.26		−13.24
10	2	8	0.75	0.476		13.03		−14.65
9	3	8	0.75	0.482		12.38		−14.02
7	4	8	0.25	0.37		4.22		−18.96
4	5	10	1	0.507		8.96		−16.37
6	6	12	0.75	0.417		12.89		−14.56
1	7	6	0.5	0.442		9.32		−15.87
2	8	10	0.5	0.421		11.98		−12.81
5	9	4	0.75	0.509		16.68		−8.59
8	10	8	1.25	0.554		6.21		−15.44
3	11	6	1	0.519		15.24		−8.97

## Data Availability

Data is contained within the article and supplementary material.

## Supplementary Materials

The following supporting information can be downloaded at: https://www.mdpi.com/article/10.3390/ph16091311/s1, Figure S1: Green synthesis of gold nanoparticles (a) initial color (b) color change after 1 h of incubation; Figure S2: Actual *vs* predicted plot for responses (A) Y_1_, (B) Y_2_ and (C) Y_3_; Figure S3: Interaction plot for responses (A) Y_1_, (B) Y_2_ and (C) Y_3_; Figure S4: Normal plots of residuals (A–C) and residual *vs* run plots (D–F) for responses Y_1_ (A and D), Y_2_ (B and E) and Y_3_ (C and F); Figure S5: Overlay contour plot; Table S1: MICs of CME and *Cord*-Au-NPs against the studied bacterial strains; Table S2: Brine shrimp lethality results of *Cor*-Au-NPs.
