# Supplementary material for: Process Optimization for the Bioinspired Synthesis of Gold Nanoparticles Using Cordyceps militaris, Its Characterization, and Assessment of Enhanced Therapeutic Efficacy
Source: Pharmaceuticals (Basel). 2023 Sep 16;16(9):1311. doi: 10.3390/ph16091311 (PMC10537427; doi:10.3390/ph16091311)
Supplement: Supplementary file 1 [file pharmaceuticals-16-01311-s001.zip › pharmaceuticals-2577612-SI.pdf]

pharmaceuticals

Supplementary File

Process Optimization for the Bioinspired Synthesis of Gold Nanoparticles Using *Cordyceps militaris*, Its Characterization, and Assessment of Enhanced Therapeutic Efficacy

Girish Gawas [1], Muniappan Ayyanar [2], Nilambari Gurav [3], Dinesh Hase [4], Vaishali Murade [5], Sameer Nadaf [6], Mohd Shahnawaz Khan [7], Rupesh Chikhale [8], Mohan Kalaskar [9] and Shailendra Gurav [1,*]

[1] Department of Pharmacognosy, Goa College of Pharmacy, Goa University, Panaji 403001, India; girishgawas95@gmail.com
[2] Department of Botany, A.V.V.M. Sri Pushpam College (Autonomous), Affiliated to Bharathidasan University, Poondi 613503, India; asmayyanar@yahoo.com
[3] PES's Rajaram and Tarabai Bandekar College of Pharmacy, Goa University, Ponda 403401, India; nilagurav@rediffmail.com
[4] Department of Pharmacognosy, Amrutwahini College of Pharmacy, Sangamner 422608, India; dinesh23787@gmail.com
[5] Department of Chemistry and Research Centre, Padmashri Vikhe Patil College Pravaranagar, Loni 445001, India; vaishali.hase66@gmail.com
[6] Bharati Vidyapeeth College of Pharmacy, Palus 416310, India; sam.nadaf@rediffmail.com
[7] Department of Biochemistry, College of Science, King Saud University, Riyadh 11451, Saudi Arabia; moskhan@ksu.edu.sa
[8] UCL School of Pharmacy, 29–39 Brunswick Square, London WC1N 1AX, UK; r.chikhale@ucl.ac.uk
[9] R. C. Patel Institute of Pharmaceutical Education and Research, Shirpur 425405, India; kalaskar.mohan@gmail.com
* Correspondence: shailendra.gurav@nic.in; Tel.: +91-99-7083-5019

Abstract: The promising therapeutic implications of nanoparticles have spurred their development for biomedical applications. An eco-friendly methodology synthesizes gold nanoparticles using *Cordyceps militaris*, an edible mushroom (*Cord*-Au-NPs), using a quality-by-design approach (central composite design). UV-visible spectroscopy analysis revealed an absorption peak in the 540-550 nm, thus confirming the synthesis of gold nanoparticles. *Cord*-Au-NPs have a crystalline structure, as evidenced by the diffraction peaks. The zeta potential value of -19.42 mV signifies the stability of *Cord*-Au-NPs. XRD study shows gold facets and EDX analysis revealed a strong peak of spherical nanoparticles in the gold region with a mean particle size of 7.18 nm and a polydispersity index of 0.096. The obtained peaks are closely associated with phenolic groups, lipids, and proteins, as examined by FTIR, suggesting that they function as the reducing agent. *Cord*-Au-NPs exhibited dose-dependent antioxidant, antidiabetic, and antibacterial activity. The method is eco-friendly, non-toxic, less time-consuming, and does not use synthetic materials, leading to higher capabilities in biomedical applications.

Keywords: green synthesis; central composite design; antioxidant activity; antidiabetic activity; process optimization

Supplementary File

2.4 Physicochemical characterization

2.4.1 UV- Visible Spectroscopy

The synthesis of *Cord*-Au-NPs was confirmed by taking an absorption spectrum of the reaction mixture in the scanning wavelength range of 200 to 800nm using a UV-visible spectrophotometer (Shimadzu, UV-2700).

2.4.2 Fourier Transform Infrared (FT-IR) Spectroscopy

Fourier Transform Infrared spectrophotometer (Make: Shimadzu, IR Affinity-1) was used to distinguish the bioreduction compounds involved in synthesizing *Cord*-Au-NPs. The spectrum was obtained using the KBr pellet method in the scanning range of 400 to 4000cm^{-1}.

2.4.3 X-ray Diffraction Study (XRD)

The synthesized *Cord*-Au-NPs were analyzed to examine the structure and composition using an X-ray diffractometer (XRD) (Rigaku, Ultima IV) at an angular range of 10°<2θ>80° at 40kV and 20mA having a 10mm divergence slit in 2θ/θ continuous scanning mode.

2.4.4 Scanning Electron Microscopy (SEM) and Energy Dispersive X-ray (EDX) Analysis

The microstructure and elemental composition of the *Cord*-Au-NPs were analyzed using SEM with EDX analysis (Carl ZEISS EVO 18). Magnification ranges of 1μm, 2μm, and 200nm were used to observe and visualize the *Cor*-ZnONPs morphology during SEM.

2.4.5 High-Resolution Transmission Electron Microscopy (HRTEM) and Selected Area Electron Diffraction (SAED)

HRTEM analysis and SAED studies of the *Cord*-Au-NPs were carried out using Transmission Electron Microscope (Make: Joel, Model: JEM 2100) to determine the morphology of the *Cor*-ZnONPs.

2.4.6 Particle Size Analysis (PSA) and Zeta Potential Analysis

The average particle size, polydispersity index, mobility and stability profile of *Cor*-ZnONPs were ascertained by performing PSA using Nanophox, NX0088 (Sympatec, Germany).

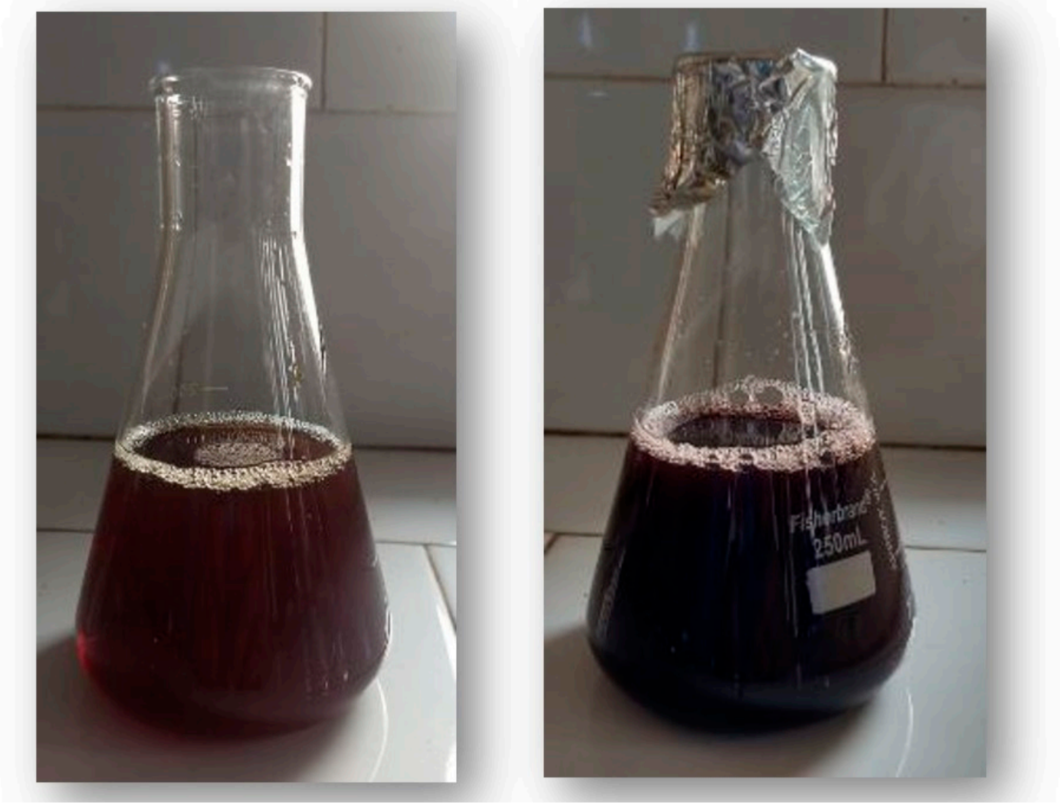

Fig. S1. Green synthesis of gold nanoparticles (a) initial color (b) color change after 1 h of incubation

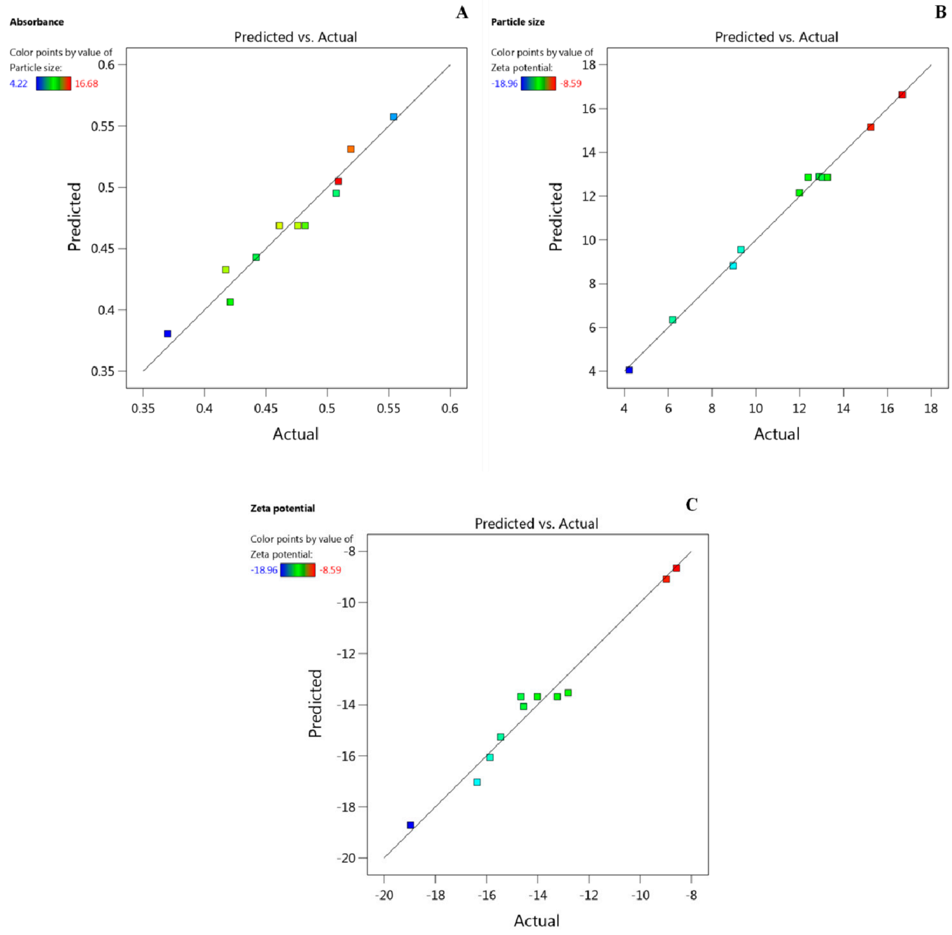

Fig. S2: Actual *vs* predicted plot for responses A) Y_1, B) Y_2 and C) Y_3

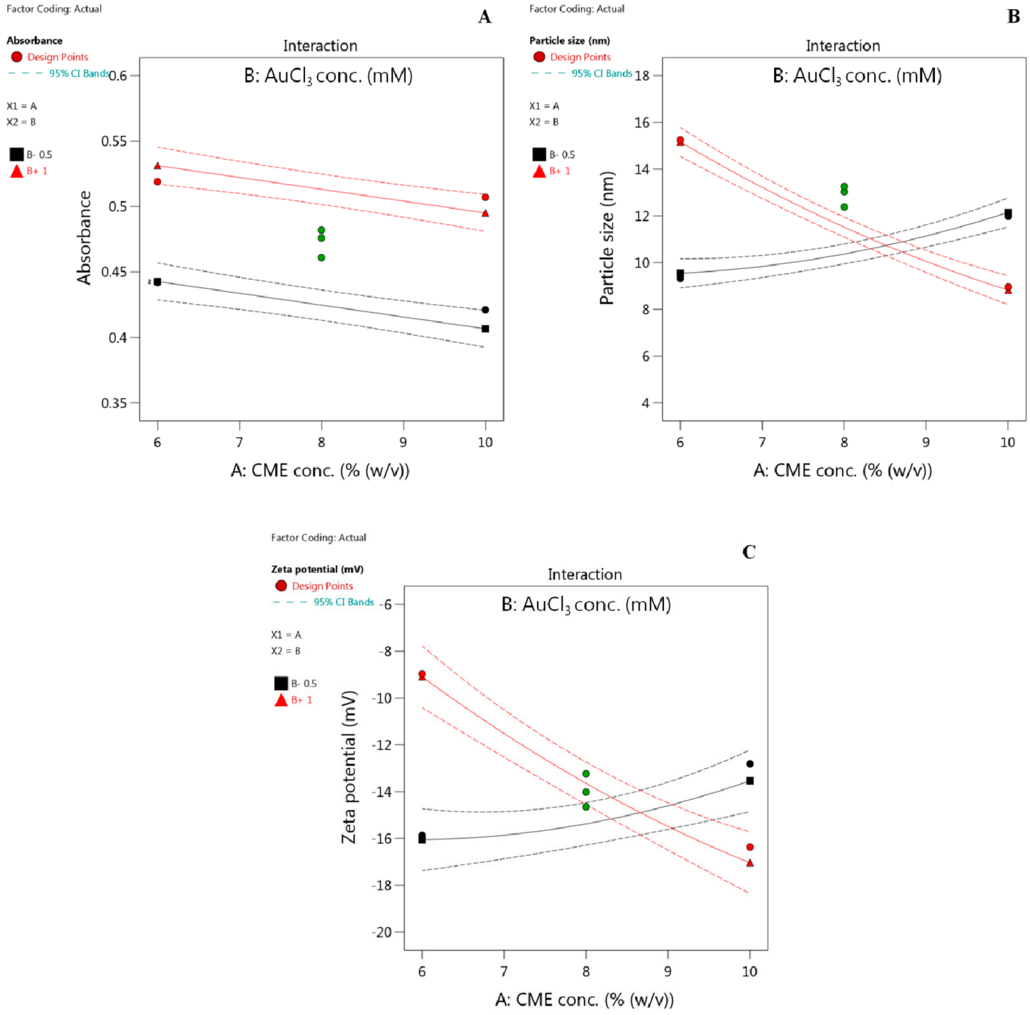

Fig. S3: Interaction plot for responses A) Y_1, B) Y_2 and C) Y_3

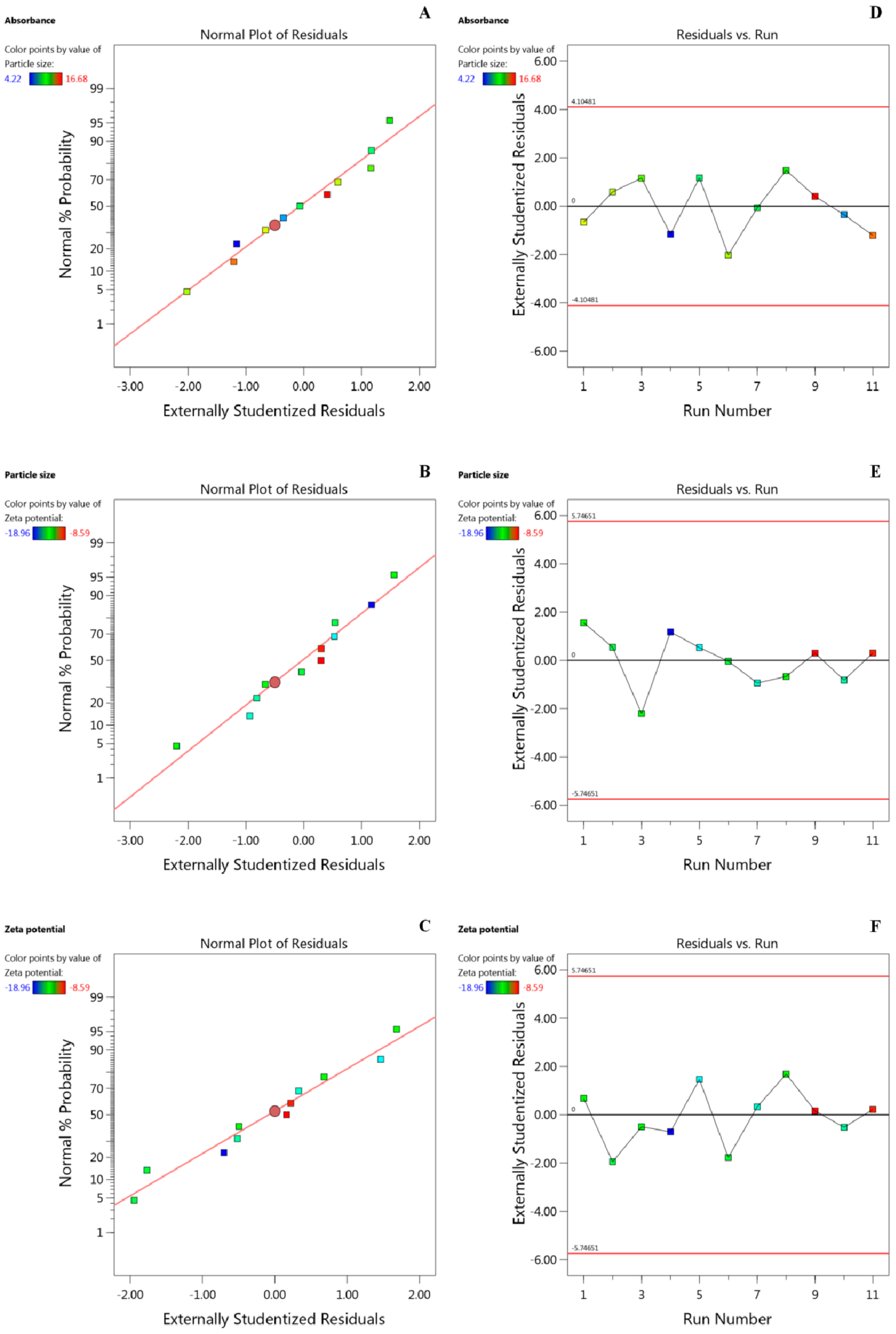

Fig. S4: Normal plots of residuals (A–C) and residual *vs* run plots (D–F) for responses Y_1 (A and D), Y_2 (B and E) and Y_3 (C and F)

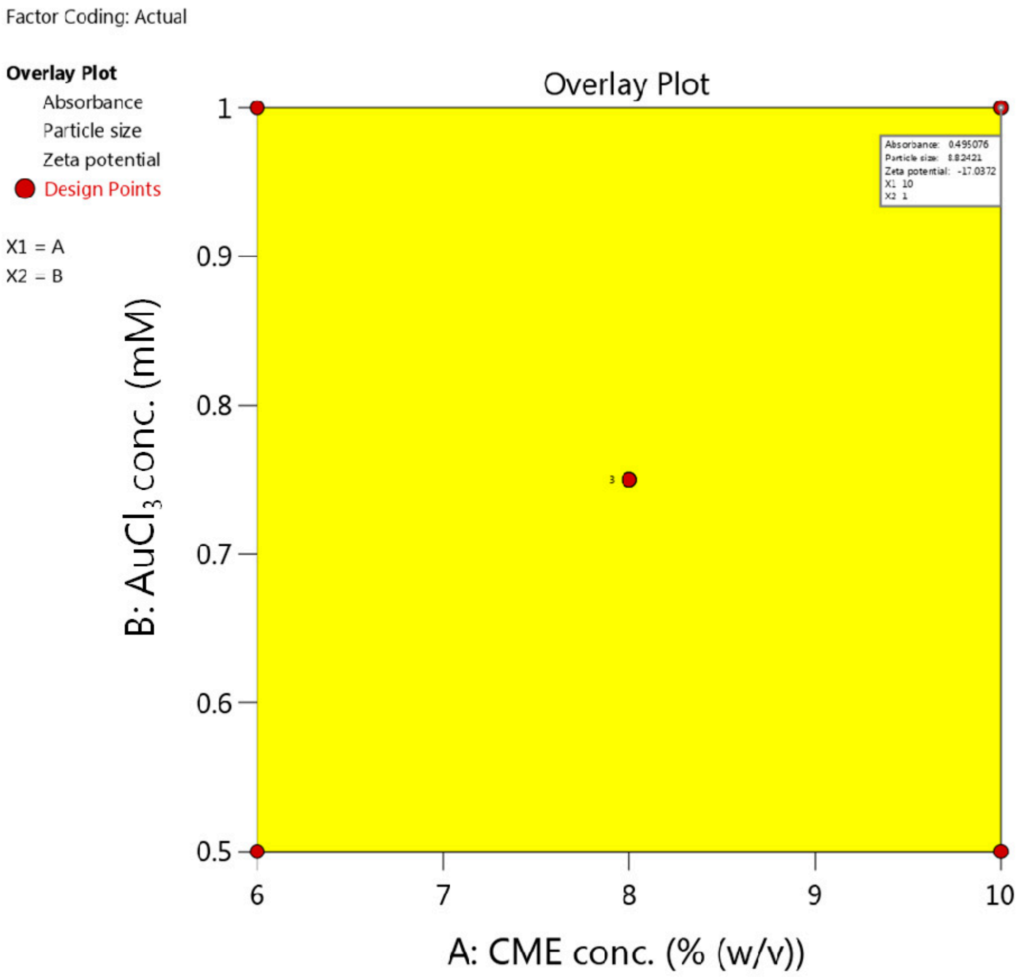

Fig. S5: Overlay contour plot

Table S1. MICs of CME and *Cord*-Au-NPs against the studied bacterial strains

Name of the organisms	MIC (μg/mL)		
	Ampicillin	CME	*Cord*-Au-NPs
Proteus vulgaris MTCC 426	15.00	46.88	23.44
Staphylococcus epidermis MTCC 435	7.50	187.50	93.75
Bacillus subtilis MTCC 441	15.00	187.50	23.44
Rhodococcus equi MTCC 2558	15.00	93.75	46.88
Shigella flexneri MTCC 1457	30.00	187.50	93.75
Pseudomonas aeruginosa MTCC 1748	7.50	93.75	46.88

Table S2: Brine shrimp lethality results of *Cor*-Au-NPs

Test drug	LC_{50} (μg/mL)
Cor-Au-NPs	192.23± 57.34
$KCrO_7$ solution (positive control)	<10 μg/mL
Seawater (negative control)	No death was observed
